# Breakthrough of interspecific chimerism: blocking apoptosis

**DOI:** 10.1186/s13619-021-00080-w

**Published:** 2021-05-05

**Authors:** Yiren Qin, Duancheng Wen

**Affiliations:** Ronald O. Perelman and Claudia Cohen Center for Reproductive Medicine, Weill Cornell Medicine, New York, NY 10065 USA

## Abstract

On January 28 in Nature online, Zheng et al. (Nature, 2021 (Online ahead of print)) reported that they developed an ingenious method of interspecies PSC co-culture system in vitro which unfolded interspecific cell competition. This study paves the way for discovering the mechanism of interspecific chimera and for further interspecific organogenesis between evolutionarily distant species.

## Main Text

Pluripotent stem cells (PSCs), such as embryonic stem cells (ESCs), induced pluripotent stem cells, and epiblast stem cells (EpiSCs), are very promising resources for study of mammalian embryonic development and regenerative medicine. Not only can human PSCs (hPSCs) differentiate into various types of cells, tissues and organoids in dish (Thomson et al. 1998; Spence et al. 2011; McCracken et al. 2014), but also, more intriguingly, into complex and functional chimeric organs in animals via the chimera technology (Wu et al. 2017). These humanized chimeric organs may bring novel resources and seductive prospects for clinic application in the future, and offer dynamic targeted drug screening systems in vivo. However, the efficiency of interspecific chimerism between evolutionarily distant species is very low, especially between human and other species (Wu et al. 2017; Theunissen et al. 2016).

It is well known that biological competition is a contest between winners and losers. Based on the research of Drosophila (Morata and Ripoll 1975), viable cells can be eliminated by their neighbors via cell competition. During interspecific chimera formation, cell competition between xenogenic donor cells and host cells plays a critical role. Compared with the host cells, xenogenic donor cells are less fit and relatively weaker competitors. For instance, Masaki et al. observed that when injected into mouse pre-implantation embryos and cultured, human cells did not integrate into the epiblast stage embryos and perished rapidly (Masaki et al. 2015). They speculated that hPSCs might have undergone apoptosis in xenogeneic embryos. Indeed, their later study elucidated that overexpression of the anti-apoptotic gene *BCL-2* could promote rat PSCs to contribute to mouse blastocysts and constitute interspecific adult chimeras (Masaki et al. 2016). Using the above idea, Wang et al. attested that overexpression of *BCL2L1* and *BCL-2* in hPSCs could efficiently construct human-mouse chimeras (Wang et al. 2018).

**Fig. 1:**
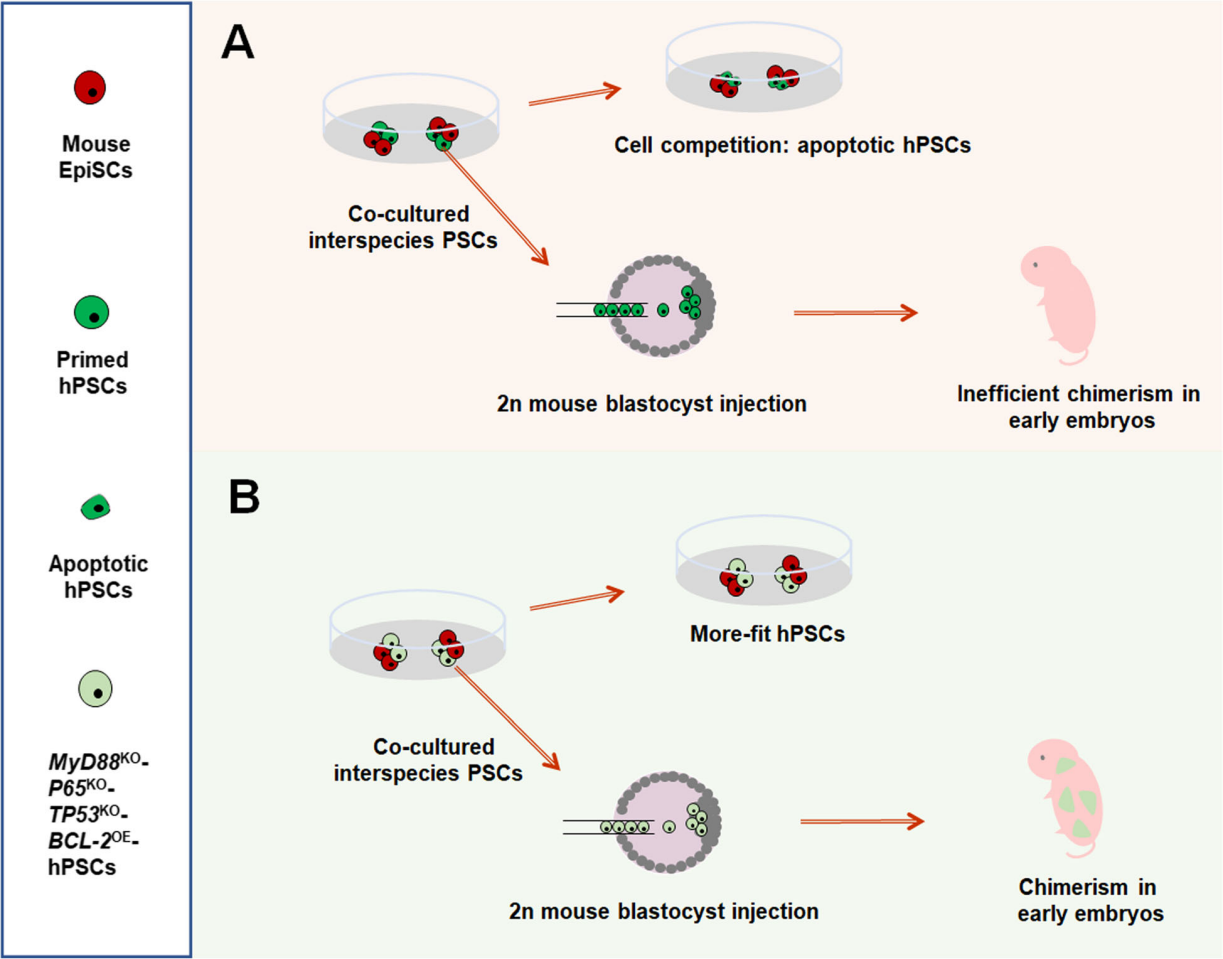
Cell competition between hPSCs and mEpiSCs and overcoming of interspecific competition. **a** The hPSCs underwent apoptosis in co-culture system of hPSCs and mEpiSCs and did not integrate into the early mouse embryos in vivo. **b** *TP53*^KO^, *P65*^KO^*, MyD88*^KO^ and *BCL-2*^OE^ hPSCs, respectively, displayed higher survival rates in the co-culture system with mEpiSCs in vitro and in early mouse embryos in vivo

Previously, Wu and his colleagues demonstrated that, unlike naïve hPSCs, the intermediate hPSCs could contribute to chimeras and produce differentiated descendants in post-implantation pig embryos (Wu et al. 2017). On the basis of this study, they recently established an ingenious method of interspecies PSC co-culture system to unfold interspecific cell competition (Zheng et al. 2021 (Online ahead of print)). They found that when primed hPSCs and mouse EpiSCs (mEpiSCs) were co-cultured in a dish, only hPSCs went through apoptosis (Fig. 1a), which was caused by cell competition through direct cell contact rather than secreted factors. Besides, these hPSCs could not integrate into the early mouse embryos (E8–9). In addition, they illustrated that the phenomenon of apoptosis also existed in other distant interspecies PSC co-culture systems, such as those of primate-rodent, primate-cow and rodent-cow, but did not exist in the proximal interspecific rat-mouse and human-rhesus co-culture systems.

Considering activation of apoptosis presents the main mechanism of less-fit cell elimination, Wu and his colleagues explored new approaches other than overexpression of *BCL-2* to explain cell competition. Firstly, they wanted to detect whether pro-apoptotic gene *TP53* was concerned in hPSC apoptosis in the human-mouse co-culture system. As expected, *TP53*^KD^ and *TP53*^KO^ hPSCs, after being co-cultured with mEpiSCs respectively, displayed higher survival rates. Secondly, in order to get more foresight about cell competition mechanism, Wu and his colleagues collected co-cultured and separately cultured hPSCs of days 1–3 to conduct RNA-sequencing. By comparative transcriptome analysis, they found that P53 and NF-κB signaling pathway-related genes were significantly upregulated in co-cultured hPSCs compared with those in separately cultured hPSCs. According to this clue, after representative genes in these pathways, such as *P65*, *MYD88* and the above preconceived *TP53* of hPSCs, were knocked out, both the survival of hPSCs in the co-culture system with mEpiSCs in vitro and the chimerism of hPSCs in E8–9 mouse embryos in vivo were significantly promoted (Fig. 1b). Furthermore, in co-culture systems between more distant species, such as those of primate-rodent, primate-cow and rodent-cow, survival rates of PSCs with apoptosis-related genes knocked out were all prominently increased.

In summary, Wu and his colleagues have exploited an ingenious co-culture system of interspecies PSCs in vitro to probe cell competition. Not only does this study pave the way for discovering mechanism of interspecific chimera, but also promotes interspecific organogenesis between evolutionarily distant species to obtain human organs in animals for transplant in the future.
